# A Case Review of Wound Bed Preparation in an Infected Venous Leg Ulcer Utilizing Novel Reticulated Open Cell Foam Dressing with Through Holes during Negative Pressure Wound Therapy with Instillation

**DOI:** 10.7759/cureus.3504

**Published:** 2018-10-27

**Authors:** Elizabeth McElroy, Stormy Lemay, Kersten Reider, Amir B Behnam

**Affiliations:** 1 Wound, Ostomy, Continence Care / Nursing Administration, Reading Health System, Reading, USA; 2 Plastic Surgery, Reading Hospital, Wyomissing, USA

**Keywords:** negative pressure wound therapy, instillation, wound cleansing, wound bed preparation, thick exudate, venous leg ulcer

## Abstract

Chronic venous insufficiency (CVI) and venous leg ulcers (VLUs) have major financial implications for patients and healthcare professionals. VLUs, in particular, require significant care, can be slow to heal, and have a high rate of recurrence. These factors combine to make VLUs a major burden on the healthcare system. Recent estimates show that the cost of treatment of VLUs per patient in the United States is $10,000 to $12,000 per year, with the average lifetime cost of care greater than $40,000. Infected VLUs often require surgical debridement for the removal of bacterial burden and biofilm. The use of negative pressure wound therapy with instillation and dwell (NPWTi-d) has shown to decrease OR visits, length of hospitalization, and therapy days in lower extremity and trunk wounds. In 2017, a novel reticulated open cell foam dressing with through holes (ROCF-CC) was introduced as a dressing option with NPWTi-d. ROCF-CC assists in removing thick wound exudate and infectious materials. This dressing option is especially helpful for wound cleansing when debridement is not possible or appropriate in patients.

## Introduction

There is an increasing demand on our healthcare system for the diagnosis of venous leg ulcers (VLU). Chronic venous insufficiency (CVI) and VLUs also have major financial implications for patients and healthcare professionals. VLUs, in particular, require significant care, can be slow to heal, and have a high rate of recurrence. These factors combinedly make VLUs a major burden on the healthcare system. Recent estimates show that the cost of treatment of VLUs per patient in the United States is $10,000 to $12,000 per year, with the average lifetime cost of care greater than $40,000 [1]. VLUs are very common, accounting for between 60% and 80% of all leg ulcers. These ulcers often require significant treatment and can lead to longer hospital stays and recurring complications. VLUs can also be debilitating and dangerous for patients and can lead to reduced mobility, severe pain, infection, amputation, and death [2]. VLUs often are contaminated with bacteria or have a biofilm on the wound base. When the bacterial burden reaches a point the patient’s immune system cannot control, infection often sets in. Decreasing the bacterial burden is important for the overall management of the wound. Negative pressure wound therapy (NPWT) can remove the exudate and infectious material from the wound base. Advances in the technology of NPWT now allow clinicians to utilize NPWT with instillation and dwell (NPWTi-d) to both dilute and solubilize the infectious materials and debris and remove them from the wound bed. Infected VLUs often require surgical debridement for the removal of bacterial burden and biofilm. The use of NPWTi has shown to decrease OR (operating room) visits, length of hospitalization, and therapy days in treating lower extremity and trunk wounds [3-4]. In 2017, a novel reticulated open cell foam dressing with through holes (ROCF-CC) was introduced as a dressing option along with NPWTi-d. ROCF-CC assists in removing thick wound exudate and infectious materials. This dressing option is especially helpful for wound cleansing when debridement is not possible or appropriate in the patients [5-7].

## Case presentation

A 60-year-old female patient was admitted to the hospital with complaints of shortness of breath. She was noted to meet the septic shock criteria as demonstrated by hypoxia, tachycardia, hypotension, and not responding to fluids. She has a past medical history of hypertension, hypothyroidism, gastroesophageal reflux disease (GERD), VLUs, and ambulatory dysfunction. She has a past surgical history of a back surgery, fracture surgery, hysterectomy, and partial thyroidectomy. She required intubation in the emergency room for her respiratory distress and was transferred to the medical intensive care for the ongoing medical care. Upon skin assessment, she was found to have a large ulcer on the distal aspect of her right lower extremity (RLE). The majority of the wound base was covered in a fibrotic slough. Her leg was shaped like an upside-down champagne bottle with pitting and woody edema. The primary team consulted both the wound, ostomy, and continence nurses (WOCN) team and plastic surgery for evaluation and treatment of the wound. While the surgical team was consulted, the primary team also noted that she was not medically stable for discharge as she was hemodynamically unstable. 

**Figure 1:**
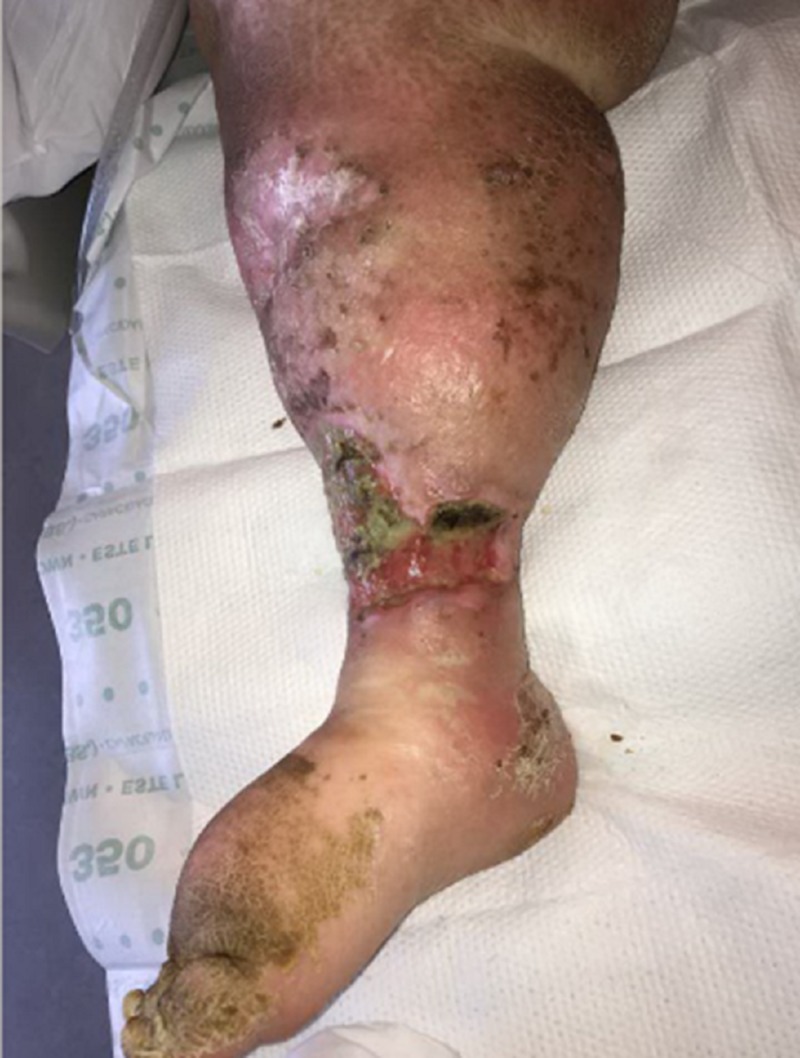
Initial presentation of the right lower extremity

**Figure 2:**
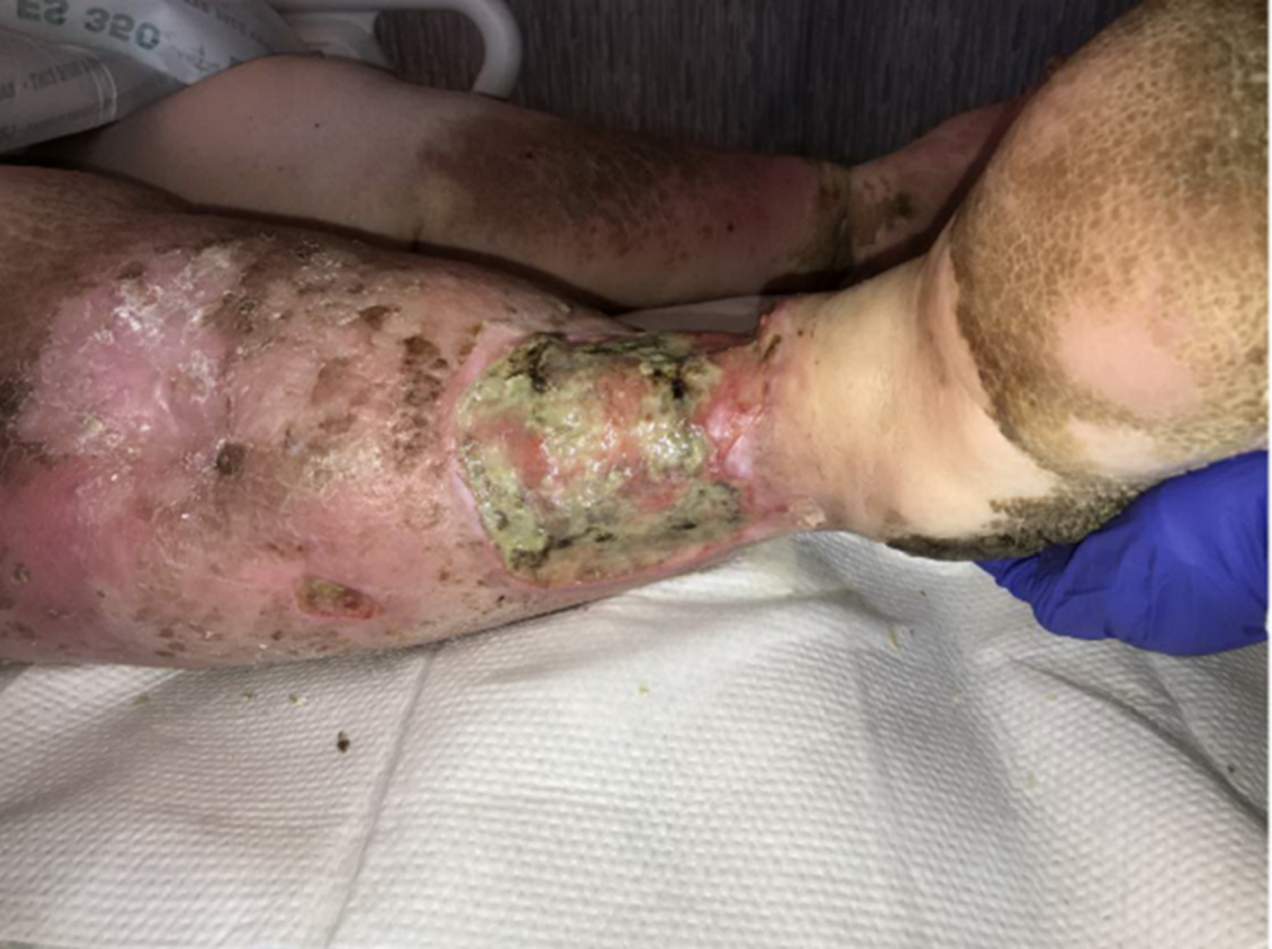
Initial presentation of the right lower extremity: lateral aspect

Upon primary assessment of the WOCN team, the patient was noted to have two ulcerations on the RLE with the larger distal ulceration measuring 10 x 16 x 1.5 cm (240 cm³) with 20 percent brown tissue, 40 percent pink tissue, and 40 percent tan tissue in the base (Figures 1, 2). There was a malodor noted as well. She was being treated with intravenous cefepime, clindamycin, and vancomycin, pending wound culture results as per the primary medical team. 

**Figure 3:**
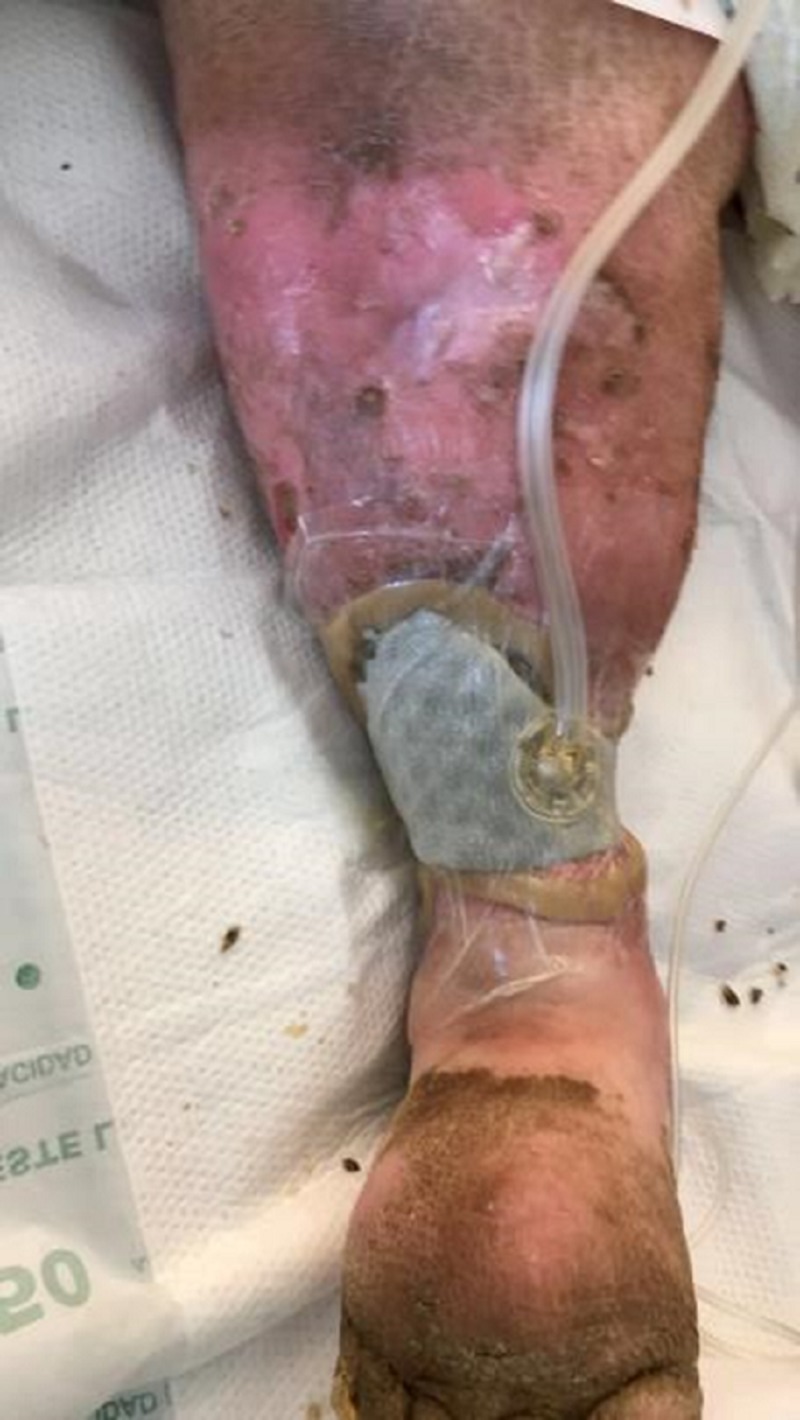
Dressing application of NPWTi-d with ROCF-CC NPWTi-d: negative pressure wound therapy with instillation and dwell; ROCF-CC: reticulated open cell foam dressing with through holes

Within 24 hours of admission, the WOCN team applied a novel ROCT-CC on to the wound base with NPWTi-d (Figure 3). The instillation solution chosen was hypochlorite solution (Dakin’s) 0.125% for the first 24 hours. The team utilized ostomy barrier rings surrounding the wound edge to facilitate a good seal and prevent periwound maceration. The settings for NPWTi-d were as follows: 34 mL of solution, dwell 10 minutes, and -125 mmHg every one hour. The arterial brachial index (ABI) revealed a normal arterial blood flow to the foot. X-ray and erythrocyte sedimentation rate (ESR) were ordered to evaluate for a deeper infection, given the close proximity to the tibia. The X-ray and ESR were normal. The following day, the solution was changed to a normal saline solution, and the machine was checked for any alarms. No alarms were noted, and the solution volume remained at 34 mL.

**Figure 4:**
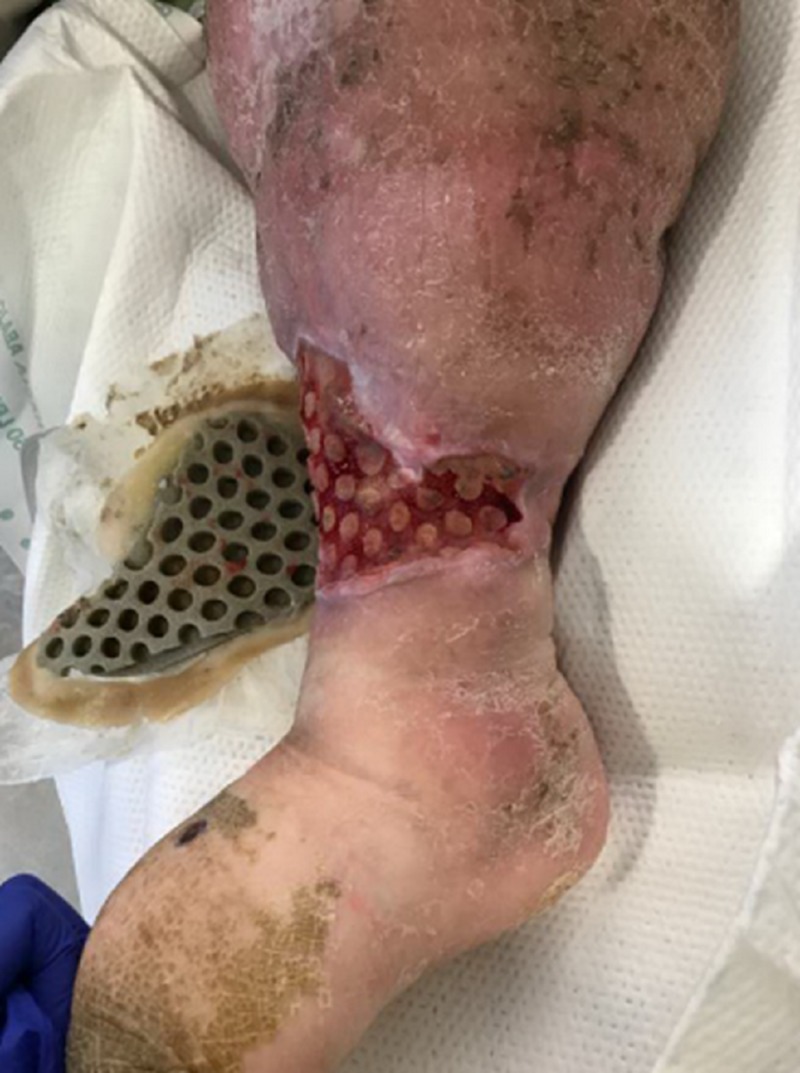
First dressing change on DOT #2; note the macrocolumns in the wound base DOT: day of treatment

**Figure 5:**
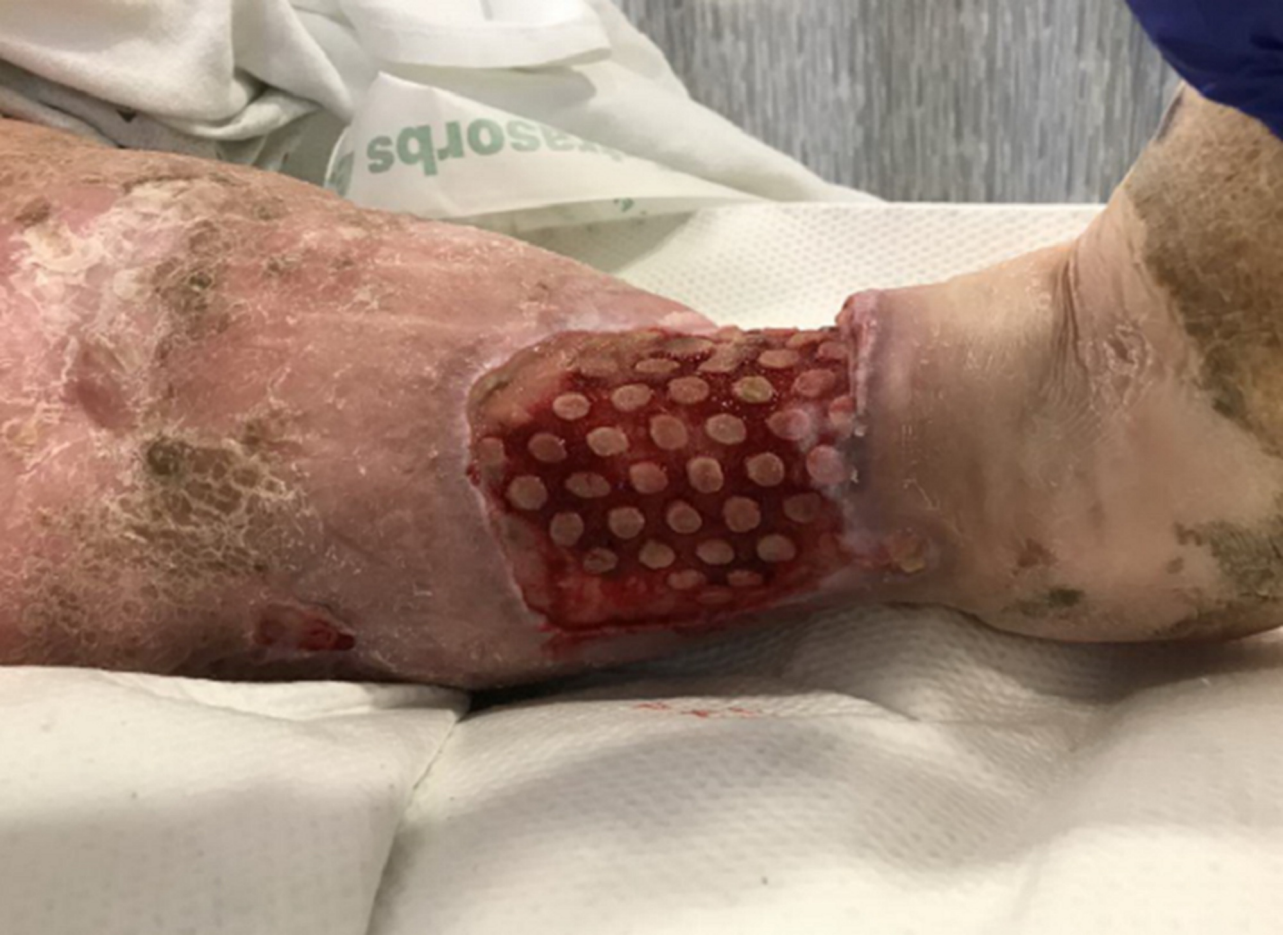
Lateral aspect after first dressing change

On the day of treatment (DOT) #2, the plastic surgeon met the WOCN team at the bedside to evaluate the wound for a surgical debridement. While the patient remained in the Medical Intensive Care Unit (MICU), she was off vasopressors and her respiratory status was improved. She was deemed medically stable for the OR. Her culture results showed *Streptococcus agalactiae* (Group B Strep) and *Proteus vulgaris*. Cefepime was stopped due to a rash, and levofloxacin was started. Upon removal of the first ROCF-CC dressing, the wound base demonstrated a rapid improvement in the granulation tissues (Figures 4, 5). The plastic surgeon evaluated the wound and deemed no surgical intervention was required at this time and that NPWTi-d should continue with ROCF-CC. The patient would be evaluated in one week for wound closure with grafting.

**Figure 6:**
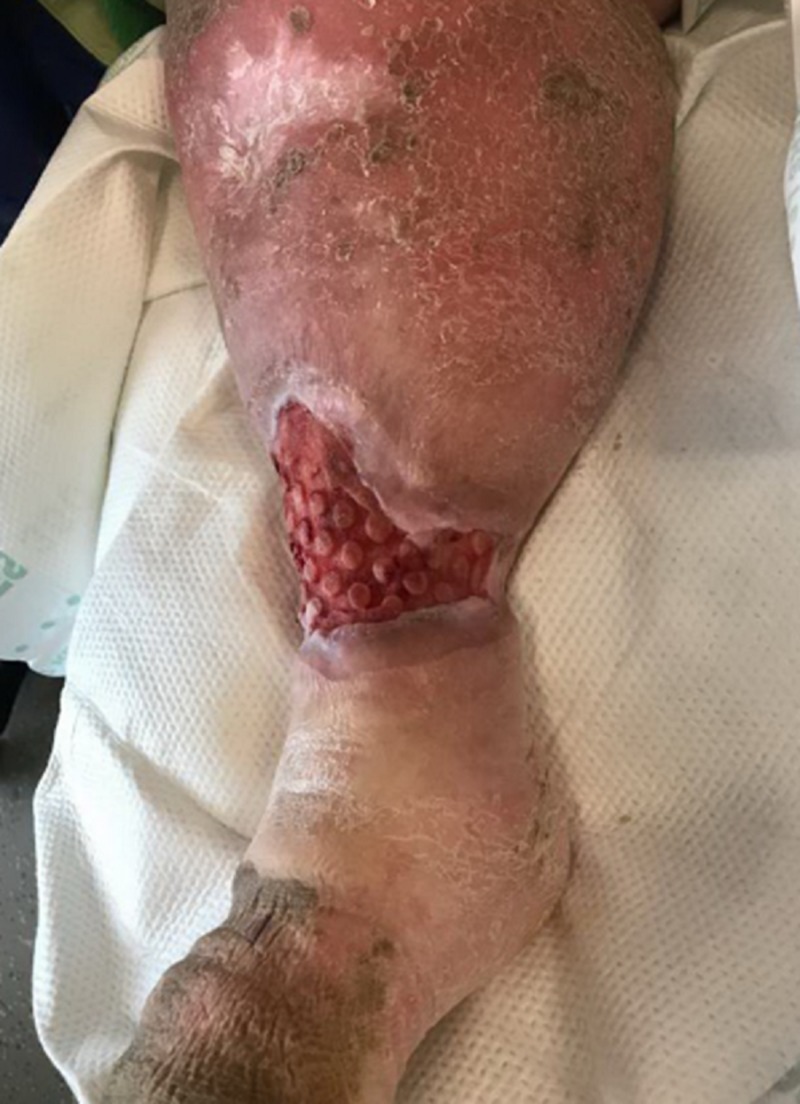
Second dressing change using NPWTi-d with ROCF-CC NPWTi-d: negative pressure wound therapy with instillation and dwell; ROCF-CC: reticulated open cell foam dressing with through holes

The patient continued with routine dressing changes every 48-72 hours. On the eighth day of therapy, the patient was transitioned to NPWTi-d with ROCF (not ROCF-CC) for an additional two days (Figure 6). The decision to transition to the different dressing was due to the lack of a thick exudate or non-viable tissue in the wound base. 

**Figure 7:**
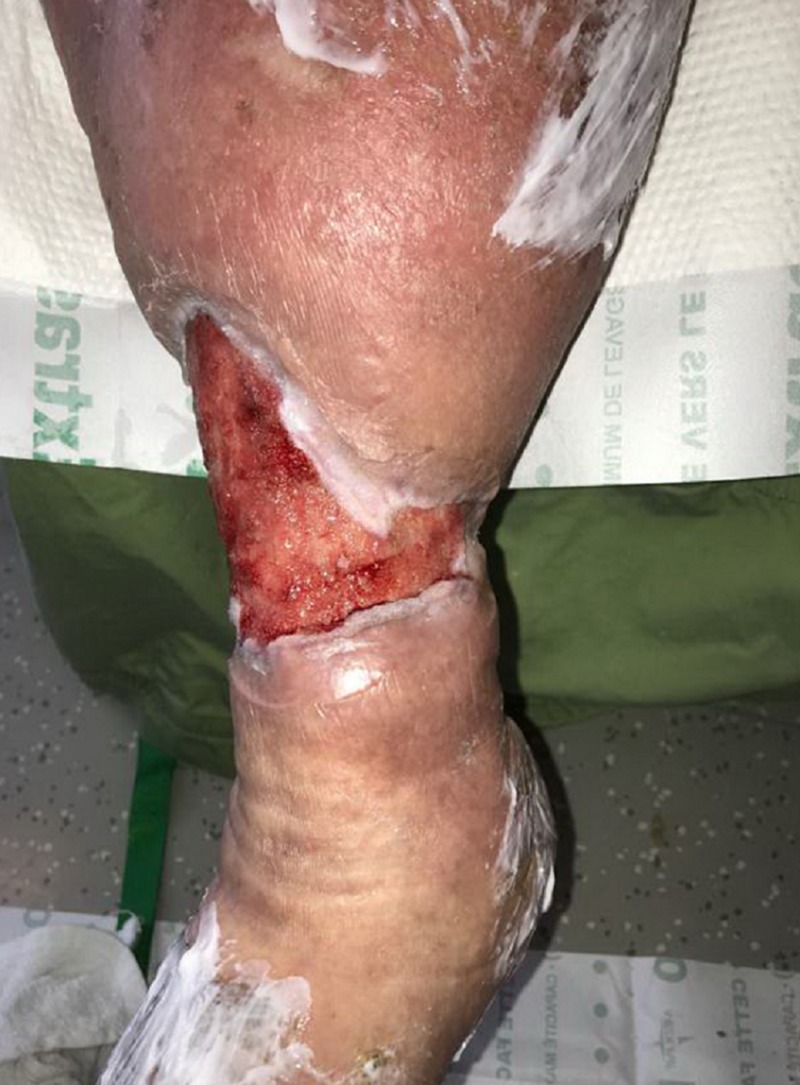
Appearance of right lower extremity after use of NPWTi-d; patient transitioned to local wound care with collagen dressing with ORC and silver alginate NPWTI-d: negative pressure wound therapy with instillation and dwell; ORC: oxidized regenerated cellulose;

**Figure 8:**
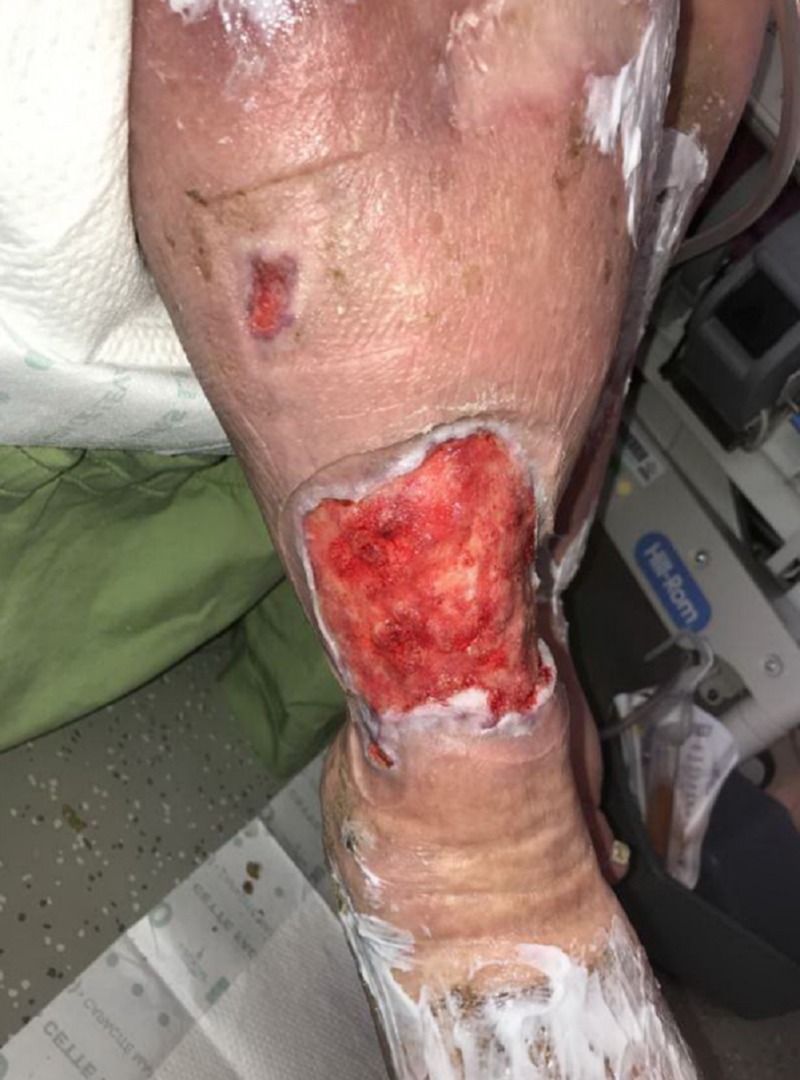
Wound bed appearance after use of NPWTi-d; note the robust granulation tissue in the base NPWTi-d: negative pressure wound therapy with instillation and dwell

At the next dressing change on DOT#10, the patient was transitioned from NPWTi-d to local wound care with a collagen dressing with oxidized regenerated cellulose (ORC), a silver alginate with non-adherent contact layer, and multiplayer compression therapy for management of lymphedema (Figures 7, 8). 

**Figure 9:**
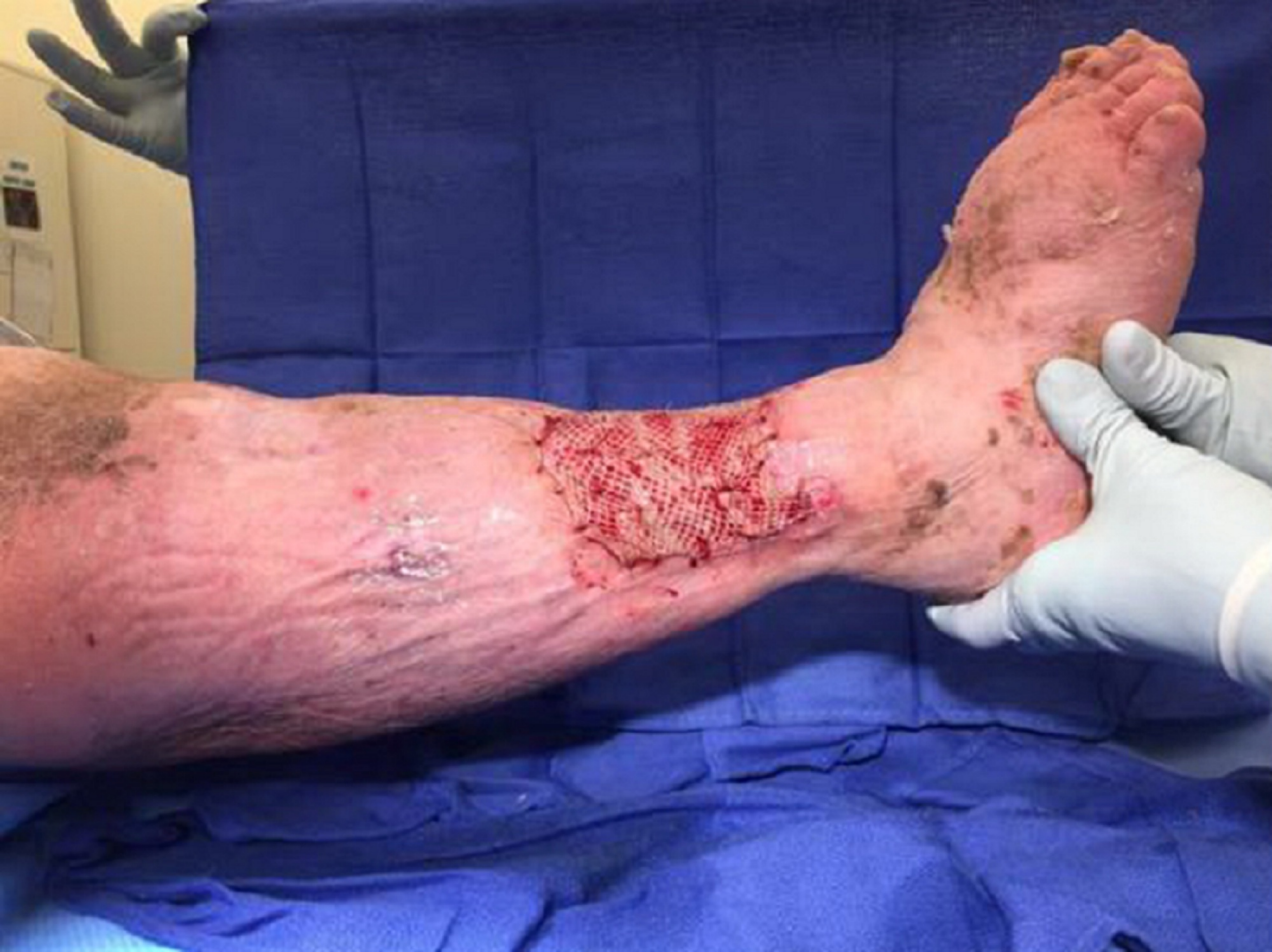
Allograft application in the OR, lateral aspect OR: operating room

**Figure 10:**
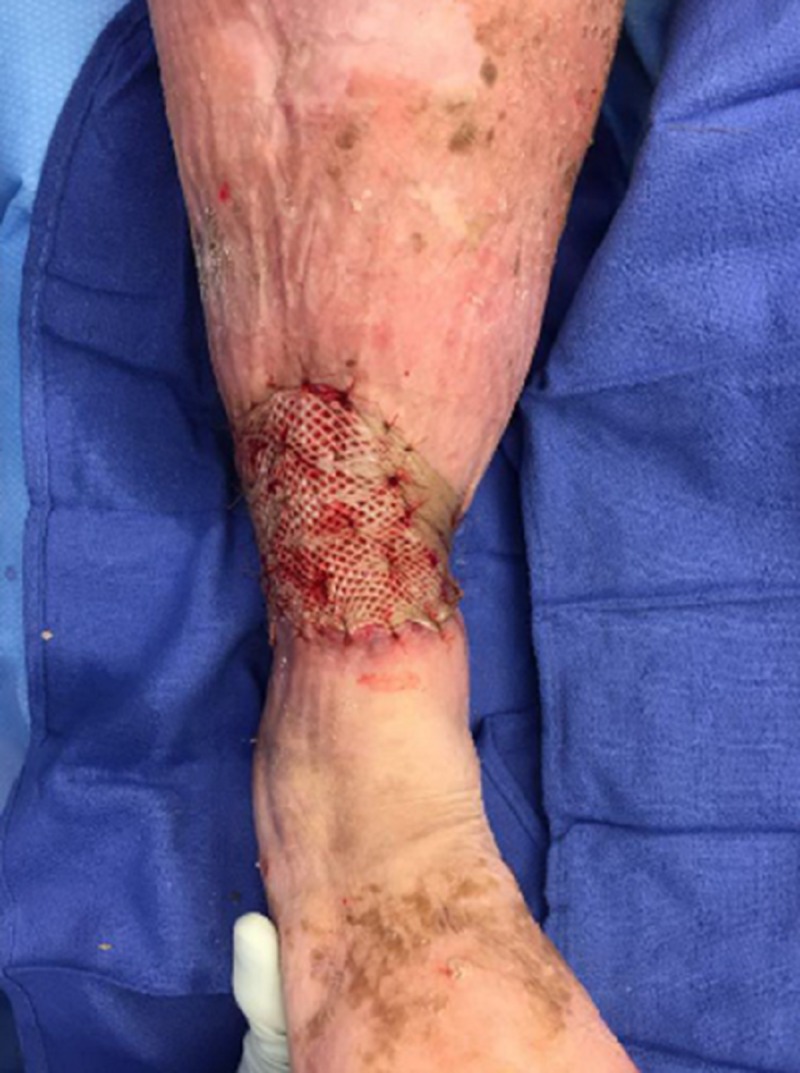
Anterior view of allograft after being applied

**Figure 11:**
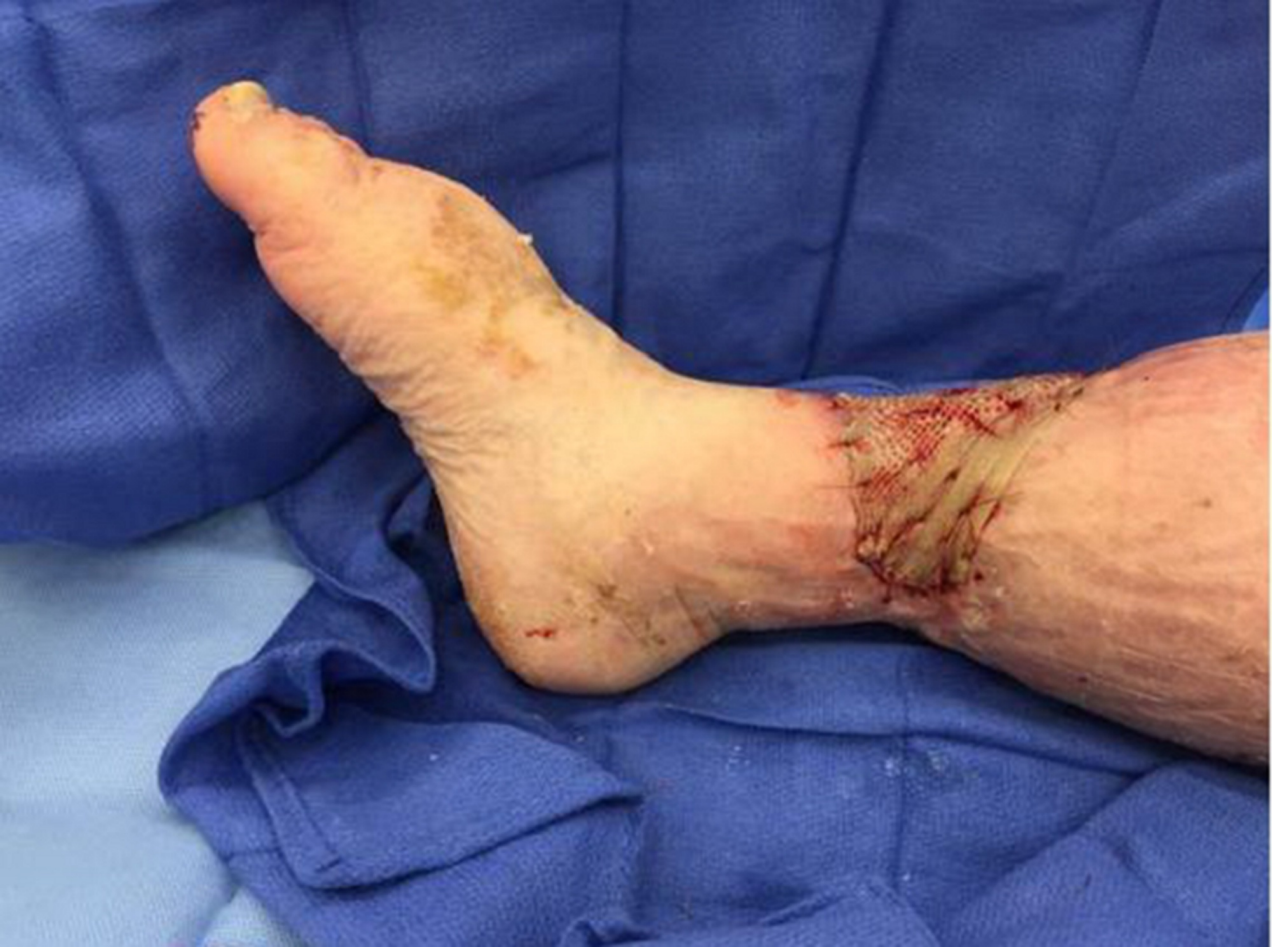
Medial aspect of the wound after allograft application

This dressing was changed every 48-72 hours over the next 11 days until the plastic surgeon applied an allograft to the wound base (Figures 9-11). An absorptive secondary dressing was applied over the graft, and multi-layer compression was reapplied.

The patient was then discharged from the acute care hospital to a skilled nursing facility (SNF) for rehabilitation services and the ongoing wound and lymphedema care. 

The patient spent 23 days in the acute care hospital before being discharged to the SNF. She underwent no operative debridement, three applications of NPWTi-d with ROCF-CC, one application of NPWTi-d with ROCF, five applications of local wound care (utilizing a collagen matrix dressing with ORC and silver alginate and multiplayer compression), and then closure with the use of an allograft.

## Discussion

VLUs present a huge financial burden to the healthcare system and can be recalcitrant to the traditional therapies. The use of advanced wound therapies is imperative to achieve wound closure and prevent infection, re-admission, sepsis, or limb loss. Especially in chronic wounds, initiating advanced wound therapies early in the treatment of the wounds is of utmost importance. It has been well established that surgical debridement is the gold standard for the removal of the bacterial burden of chronic wounds. However, surgical debridement is not always a viable option. This case study reflects that NPWTi-d may present a viable option for patients when surgical or operative debridement is not an immediate option, but the removal of a thick exudate or a non-viable tissue is critical for optimal wound healing. This option also reflects an option for immediately cleansing the wound in a controlled environment that can be performed by a multitude of clinicians, including the nursing staff, medical staff, residents, or the surgical staff. The ability of the interdisciplinary team to apply this dressing prevents a delay in starting advanced wound therapies.

The authors recognize that this is a single case study and that the implications for practice cannot be generalized. This does, however, provide an opportunity for further research to see what settings, patients, and clinical diagnosis may benefit the most from the advanced wound therapy of NPWTi-d with ROCF-CC.

## Conclusions

NPWTi-d currently offers additional dressing options that can expand the ability of the clinician to treat thick exudates, bacterial burden, and non-viable tissues in conjunction with or as an alternative to a surgical procedure. In this case, the use of NPWTi-d with ROCF-CC allowed for rapid wound bed preparation without the patient to have to undergo a surgical procedure.
